# Associations between *H19* polymorphisms and neuroblastoma risk in Chinese children

**DOI:** 10.1042/BSR20181582

**Published:** 2019-04-05

**Authors:** Chao Hu, Tianyou Yang, Jing Pan, Jiao Zhang, Jiliang Yang, Jing He, Yan Zou

**Affiliations:** 1 Department of Pediatric Surgery, Guangzhou Women and Children’s Medical Center, Guangzhou Medical University, Guangzhou 510623, Guangdong, China; 2 Department of Pediatric Surgery, Guangzhou Institute of Pediatrics, Guangzhou Women and Children’s Medical Center, Guangzhou Medical University, Guangzhou 510623, Guangdong, China; 3 Department of Pediatric Surgery, The First Affiliated Hospital of Zhengzhou University, Zhengzhou 450052, Henan, China

**Keywords:** H19, long noncoding RNA, neuroblastoma, SNP

## Abstract

**Background** *H19* polymorphisms have been reported to correlate with an increased susceptibility to a few types of cancers, although their role in neuroblastoma has not yet been clarified.

**Materials and methods** We investigated the association between three single polymorphisms (rs2839698 G>A, rs3024270 C>G, and rs217727 G>A) and neuroblastoma susceptibility in Chinese Han populations. Three hundred ninety-three neuroblastoma patients and 812 healthy controls were enrolled from the Henan and Guangdong provinces. Odds ratios (ORs) and 95% confidence intervals (CIs) were used to determine the strength of the association of interest.

**Results** Separated and combined analyses revealed no associations of the rs2839698 G>A, rs3024270 C>G or rs217727 G>A polymorphisms and neuroblastoma susceptibility. In the stratification analysis, female children with rs3024270 GG genotypes had an increased neuroblastoma risk (adjusted OR = 1.61, 95% CI = 1.04–2.50, *P*=0.032).

**Conclusion** The rs3024270 GG genotype might contribute to an increased neuroblastoma susceptibility in female Chinese children.

## Background

Neuroblastoma is the most common malignant extracranial solid tumor in children, accounting for 7–10% of all tumors in children [1,2]. Neuroblastoma originates from neural crest precursor cells of the sympathetic nervous system and is mainly located on the adrenal medulla, paraspinal ganglia, and sympathetic trunk [3–5]. The outcome of neuroblastoma is affected by several factors, such as age of onset, pathology subtypes, International Neuroblastoma Staging System (INSS) stage, c-Myc status, DNA ploidy, and structural chromosomal aberrations [3–5].

Genetic factors are critically important in neuroblastoma tumorigenesis. Approximately 1% of patients have a family history of neuroblastoma, and these patients are carriers of certain gene mutations. For example, the anaplastic lymphoma kinase (*ALK*) gene and paired-like homeobox 2b (*PHOX2B*) gene have been identified as predisposing factors to familial neuroblastoma [6–8]. Evidence from genome-wide association studies (GWASs) of sporadic cases also suggests that genetic factors might be involved in the pathogenesis of neuroblastoma [9,10]. These studies indicate an important role of genetic characteristics in the tumorigenesis of neuroblastoma. Single nucleotide polymorphisms (SNPs) or other gene mutations, such as rare mutations in the *ALK* gene at locus 2p23 for familial neuroblastoma, common SNPs in cancer susceptibility candidate 15 (*CASC15*) and neuroblastoma-associated transcript 1 (*NBAT1*) at 6p22 were found to be involved in tumor initiation of neuroblastoma, and the bos taurus BRCA1 associated RING domain 1 (*BARD1*) gene at 2q35, *IL3*, cholesterol-lowering factor (*CFL*), and lens intrinsic membrane (*LIM*) domain only 1 (*LMO1*) were found to be related to the risk of sporadic neuroblastoma.

*H19* is a paternally imprinted gene that is located at chromosome 11p15.5 near the *IGF2* gene. The expression of H19 peaks during embryogenesis and is down-regulated after birth in most tissues. Increasing evidence has revealed that H19 plays a critical role during tumorigenesis. H19 has been found to re-express during tumorigenesis in many kinds of tumors, such as breast cancer [11], lung cancer [12], invasive cervical carcinomas [13], esophageal cancer [14], and bladder cancer [15]. Smoking, exposure to the carcinogens, nitrosamine and diethylnitrosamine, and hypoxia are risk factors for H19 overexpression [16]. The up-regulation of *H19* enhances resistance of cancer cells to stress, such as genome destabilization and hypoxia [17].

Recently, a GWAS revealed that SNPs in *H19* are associated with cancer susceptibility [18], and the correlation of *H19* polymorphisms and cancers were confirmed in subsequent studies [19–21]. A study on colorectal cancer reported that the SNPs rs2839698 G>A, rs3024270 C>G, and rs217727 G>A, which are located at exon 1, exon 5, and intron region of the *H19* gene, respectively, have effects on the secondary structure of *H19* [20], and these SNPs were found to be associated with an increased cancer risk in Chinese populations [20,22–27]. However, few studies have focused on neuroblastoma. Herein, we performed a two-center hospital-based case–control study using data from 393 neuroblastoma patients and 812 control subjects to evaluate the association between the *H19* gene rs2839698, rs3024270, and rs217727 polymorphisms and neuroblastoma risk in Chinese children.

## Materials and methods

### Study subjects

The subjects enrolled were described in previous studies [28,29]. Briefly, a total of 393 neuroblastoma patients and 812 cancer-free controls from two provinces of China were enrolled in the present study. Two hundred seventy-five neuroblastoma patients and 531 controls were recruited from the Guangzhou Women and Children’s Medical Center in South China, and 118 neuroblastoma patients and 281 controls were recruited from the Henan province in North China [30,31]. The diagnosis and clinical stages of neuroblastoma were made according to the INSS [32]. The healthy controls had no history of malignancies and were matched to the neuroblastoma cases in terms of age (±5 years), sex, ethnicity, and geographical region [33–35]. Both neuroblastoma patients and controls were unrelated Chinese Han individuals living in the Guangdong and Henan provinces of China. The present study was approved by the Institutional Review Board of each hospital, and written informed consent was obtained from the participants’ parents or legal guardians.

### SNP selection and genotyping

The dbSNP database and SNPinfo were used for selecting candidate SNPs. Three widely investigated SNPs (rs2839698, rs3024270, and rs217727) in the *H19* gene were studied. SNP genotyping was performed by the TaqMan real-time PCR system as previously reported [36–40]. To validate the accuracy of the genotyping results from TaqMan real-time PCR, approximately 10% of the samples were randomly selected as quality control samples and genotyped using sequencing. The concordance for the quality control samples was 100%.

### Statistical analysis

All statistical tests were two-sided, with a significance level of *P*<0.05. All statistical analyses were performed using SAS software (version 9.4; SAS Institute, Cary, NC, U.S.A.). Two-sided *χ^2^* tests were used to analyze the demographic data and genotype frequencies. The Hardy–Weinberg equilibrium was assessed by goodness-of-*χ^2^* test. Odds ratios (ORs) and 95% confidence intervals (CIs) were calculated to evaluate associations between *H19* polymorphisms and neuroblastoma susceptibility.

## Results

### Associations between H19 gene polymorphisms and neuroblastoma susceptibility

In the present study, 392 patients and 810 controls were successfully genotyped. The genotype frequencies of all the three selected SNPs were in Hardy–Weinberg equilibrium (rs2839698, *P*=0.090; rs3024270, *P*=0.159; rs217727, *P*=0.470). The genotype frequencies of the three SNPs in the patients and controls are listed in Table 1. None of the three SNPs were found to be associated with an increased neuroblastoma risk in the separate analyses of each SNP or in the combined analysis on protective genotypes of the three SNPs.

**Table 1:** Logistic regression analysis for the associations between *H19* polymorphisms and neuroblastoma risk

Genotype	Cases (*n*=393)	Controls (*n*=810)	*P*^1^	Crude OR (95% CI)	*P*	Adjusted OR (95% CI)^2^	*P*^2^
rs2839698 (HWE = 0.090)							
GG	179 (45.55)	365 (45.06)		1.00		1.00	
AG	175 (44.53)	373 (46.05)		0.96 (0.74–1.23)	0.732	0.96 (0.75–1.24)	0.754
AA	39 (9.92)	72 (8.89)		1.11 (0.72–1.70)	0.650	1.11 (0.73–1.71)	0.624
Additive			0.797	1.01 (0.84–1.22)	0.890	1.02 (0.84–1.23)	0.858
Dominant	214 (54.45)	445 (54.94)	0.874	0.98 (0.77–1.25)	0.874	0.99 (0.77–1.26)	0.901
Recessive	354 (90.08)	738 (91.11)	0.561	1.13 (0.75–1.70)	0.561	1.14 (0.75–1.71)	0.541
rs3024270 (HWE = 0.159)							
CC	99 (25.19)	213 (26.30)		1.00		1.00	
CG	203 (51.65)	424 (52.35)		1.03 (0.77–1.38)	0.842	1.03 (0.77–1.38)	0.829
GG	91 (23.16)	173 (21.36)		1.13 (0.80–1.60)	0.486	1.14 (0.80–1.61)	0.472
Additive			0.764	1.06 (0.89–1.27)	0.494	1.07 (0.89–1.27)	0.480
Dominant	294 (74.81)	597 (73.70)	0.682	1.06 (0.80–1.40)	0.683	1.06 (0.81–1.40)	0.667
Recessive	302 (76.84)	637 (78.64)	0.480	1.11 (0.83–1.48)	0.480	1.11 (0.83–1.48)	0.470
rs217727 (HWE = 0.470)							
GG	186 (47.33)	382 (47.16)		1.00		1.00	
AG	164 (41.73)	342 (42.22)		0.99 (0.76–1.27)	0.907	0.98 (0.76–1.27)	0.888
AA	43 (10.94)	86 (10.62)		1.03 (0.68–1.54)	0.898	1.02 (0.68–1.53)	0.922
Additive			0.979	1.00 (0.84–1.20)	0.970	1.00 (0.84–1.20)	0.997
Dominant	207 (52.67)	428 (52.84)	0.956	0.99 (0.78–1.26)	0.956	0.99 (0.78–1.26)	0.932
Recessive	350 (89.06)	724 (89.38)	0.865	1.03 (0.70–1.52)	0.864	1.03 (0.70–1.52)	0.884
Combined effect of protective genotypes							
0	258 (65.65)	551 (68.02)		1.00		1.00	
1-3	135 (34.35)	259 (31.98)	0.410	1.11 (0.86–1.44)	0.410	1.11 (0.86–1.44)	0.411

1 *χ^2^* test for genotype distributions between neuroblastoma patients and cancer-free controls.

2 Adjusted for age and sex.

### Stratification analysis

We next analyzed the association of the three SNPs with neuroblastoma risk by stratification analyses (Table 2). Stratification analyses were conducted based on age (≤18 months, >18 months), sex (female, male), site of origin (adrenal gland, retroperitoneal, mediastinum, other sites), and clinical stage (I+II+4s, III+IV). We found that the increased risk associated with the rs3024270 GG variant genotype was more pronounced in females (adjusted OR = 1.61, 95% CI = 1.04–2.50, *P*=0.032). Regarding the risk genotypes, no significant associations between the 1–3 risk genotypes and neuroblastoma risk were found.

**Table 2:** Stratification analysis for association between *H19* genotypes and neuroblastoma susceptibility

Variables	rs2839698 (patient/control)		Adjusted OR^1^	*P*^1^	rs3024270 (patient/control)		Adjusted OR^1^	*P*^1^	rs217727 (patient/control)		Adjusted OR^1^	*P*^1^	Risk genotypes (patient/control)		Adjusted OR^1^	*P*^1^
	GG/AG	AA	(95% CI)		CC/CG	GG	(95% CI)		GG/AG	AA	(95% CI)		0	1-3	(95% CI)	
Age, months																
≤18	110/276	15/29	1.29 (0.66–2.50)	0.452	95/240	31/65	1.21 (0.74–1.97)	0.454	116/270	10/35	0.67 (0.32–1.39)	0.278	85/205	41/100	0.99 (0.64–1.54)	0.962
>18	243/462	24/43	1.06 (0.63–1.79)	0.830	207/397	60/108	1.07 (0.75–1.52)	0.727	234/454	33/51	1.25 (0.79–2.00)	0.342	173/346	94/159	1.18 (0.86–1.62)	0.297
Sex																
Female	148/317	20/24	1.79 (0.96–3.34)	0.068	123/278	45/63	**1.61 (1.04–2.50)**	**0.032**	152/302	16/39	0.83 (0.45–1.53)	0.546	107/239	61/102	1.35 (0.91–1.99)	0.137
Male	206/421	19/48	0.83 (0.48–1.45)	0.516	179/359	46/110	0.85 (0.57–1.25)	0.398	198/422	27/47	1.22 (0.74–2.02)	0.442	151/312	74/157	0.98 (0.70–1.37)	0.899
Sites of origin																
Adrenal gland	140/738	13/72	0.97 (0.52–1.80)	0.915	115/637	38/173	1.23 (0.82–1.84)	0.327	133/724	20/86	1.24 (0.73–2.08)	0.430	94/551	59/259	1.33 (0.93–1.90)	0.122
Retroperitoneal	79/738	8/72	1.01 (0.47–2.18)	0.982	67/637	20/173	1.08 (0.64–1.83)	0.776	82/724	5/86	0.52 (0.21–1.32)	0.169	62/551	25/259	0.85 (0.52–1.39)	0.516
Mediastinum	98/738	11/72	1.16 (0.59–2.27)	0.661	89/637	20/173	0.84 (0.50–1.40)	0.493	95/724	14/86	1.25 (0.68–2.28)	0.478	75/551	34/259	0.97 (0.63–1.50)	0.901
Others	30/738	6/72	2.00 (0.81–4.99)	0.135	24/637	12/173	1.82 (0.89–3.73)	0.100	34/724	2/86	0.50 (0.12–2.13)	0.350	22/551	14/259	1.35 (0.68–2.69)	0.391
Clinical stage																
I+II+4s	146/738	16/72	1.13 (0.64–2.00)	0.677	126/637	36/173	1.06 (0.70–1.59)	0.792	143/724	19/86	1.13 (0.66–1.91)	0.661	107/551	55/259	1.10 (0.77–1.57)	0.602
III+IV	190/738	21/72	1.17 (0.70–1.95)	0.561	160/637	51/173	1.19 (0.83–1.71)	0.335	187/724	24/86	1.06 (0.65–1.71)	0.821	135/551	76/259	1.20 (0.87–1.65)	0.259

1 Adjusted for age and sex.

Values in bold inidicate *P*<0.05.

## Discussion

In the present study, we found the *H19* gene rs3024270 C>G polymorphism was associated with neuroblastoma susceptibility in females. This is the first study to report an association between the rs3024270 GG genotype and an increased neuroblastoma risk in female Chinese children in a recessive model.

The association between these SNPs and cancer risk has been studied, while evidence of rs3024270 GG genotype and cancer risk are relatively limited. A study by Li et al. [20] reported an increased risk of colorectal cancer in a Chinese population with the rs3024270 GG genotype. A meta-analysis conducted by Lv et al. [21] investigated the association between long noncoding RNA polymorphisms and cancer risk, and the rs3024270 C>G polymorphism was reported to be correlated with an overall cancer risk. However, another study found a decreased risk of invasive bladder cancer in patients with the rs3024270 CC genotype in the Chinese population [41]. Besides the rs3024270 C>G polymorphism, a series of studies reported the role of the rs2839698 G>A polymorphism in platinum-based chemotherapy response in lung cancer [42], colorectal cancer [20], gastric cancer [24] and rheumatoid arthritis [22]. The rs217727 G>A polymorphism was also reported to be associated with a risk of oral squamous cell carcinoma [19], osteosarcoma [43], bladder cancer [44], cervical cancer of the squamous carcinomas subtype [20], and breast cancer [27]. Our study provides new evidence that the rs3024270 C>G polymorphism in the *H19* gene might be involved in the development of neuroblastoma.

SNPs studied in our research are all located in the *H19* gene and were reported to have an impact on the folding structure of *H19* [20], which may affect the expression or functions of H19. The role of H19 in cancers has been widely studied. The *H19* gene is an imprinted oncofetal gene that is located on chromosome 11p15.5 near the *IGF2* gene locus [45]. Wada et al. [46] reported a maintenance of normal imprinting of the *H19* and *IGF2* genes in neuroblastoma. The expression of H19 was significantly up-regulated in hepatic metastases of several types of cancers [47]. Growing evidence indicates that H19 is involved in both the proliferation and differentiation processes of cancer cells. H19 promotes cell cycle progression in breast cancer, while the depletion of *H19* RNA arrests breast cancer cells in the pre-S-phase of the cell cycle [48]. The knockdown of H19 inhibits the growth of HCC and gastric cancer cells under hypoxic recovery conditions [49].

The mechanisms by which these SNPs regulate H19 expression and are involved in cancer are unclear. A possible explanation is that SNPs may affect *H19* gene expression and function. It was reported that rs3024270 C>G, rs2839698 G>A, and rs217727 G>A dramatically altered the secondary structure of H19, and the rs3024270 GG genotype was associated with an increased risk of colorectal cancer in a Chinese population. H19 was found to act as a primary microRNA precursor, and H19 expression regulated specific mRNAs via post transcription [53]. Liang et al. [54] reported that lncRNA H19 modulated the expression of multiple genes involved in colorectal cancer by acting as a competing endogenous RNA, and H19 was also reported to produce antisense 91H, which increased the stability in cancer cells, leading to the accumulation of 91H in cancer cell lines and breast cancer tissues [55]. SNPs located in the introns and exons of the *H19* gene might change the promoter activity and function of H19 by the alteration of target miRNAs, such as hsa-miR-24-1-5p, hsa-miR-4486, and hsa-miR-566 [55]. Additionally, H19 was also found to interact with other tumor suppressor genes, such as *p53* and *c-Myc*. Increased expression of H19 during hypoxic conditions was found in cell lines with a mutant *p53* gene but not in cells with wild-type *p53* gene [50], and overexpression of H19 partially suppressed p53 activation in gastric cancer cells [51]. H19 expression was activated by c-Myc via direct binding to the regulatory region of the *H19* gene [52].

In our study, we found the GG allele pattern in 45/63 female NB patients and in 46/110 male NB patients. In the stratification analysis based on sex, disequilibrium of the G allele and the C allele was found. *H19* is an imprinted gene that is expressed from the maternal chromosome, but not the paternal chromosome [45]. This fact might partly explain the apparent disequilibrium of the analyzed SNPs. In the stratified analysis, no relation of H19 SNPs with the susceptibility of neuroblastoma patients, the origin of the tumor and the clinical stage of the tumor were found. In other studies, no relationship between rs3024270 and the susceptibility of cancer was found in stratified analyses, including age, sex, smoking, drinking etc. Further studies with larger sample sizes of patients with neuroblastoma and other tumors may provide more information.

There are several limitations of this case–control study. First, a potential selection bias would be induced because the enrollment of cases and controls occurred in a hospital. Therefore, further studies with larger populations would be of great value. Second, only three SNPs in the *H19* were included in the current study, and other polymorphisms, such as rs2735971 C>T, were not included. Furthermore, the biological functions of the three SNPs are not clear, so functional studies are needed to provide more evidence. The sample size is relatively small in the present study due to the difficulty in sample collection. We will continue our study on neuroblastoma and update the results in further study.

## Conclusion

In the present study, we reported the relationship between the rs3024270 GG genotype and the increased susceptibility of neuroblastoma in female Chinese children. Further functional studies on rs3024270 and studies with larger populations and different ethnicities would be valuable to clarify the role of *H19* SNPs in neuroblastoma.

## Data availability

The data used to support the findings of the present study are available from the corresponding authors upon request.

## Abbreviations

*ALK*: anaplastic lymphoma kinase
CI: confidence interval
GWAS: genome-wide association study
INSS: International Neuroblastoma Staging System
OR: odds ratio
SNP: single nucleotide polymorphism
